# Low perception of malaria risk among the Ra-glai ethnic minority in south-central Vietnam: implications for forest malaria control

**DOI:** 10.1186/1475-2875-9-23

**Published:** 2010-01-20

**Authors:** Koen Peeters Grietens, Xa Nguyen Xuan, Wim Van Bortel, Thang Ngo Duc, Joan Muela Ribera, Truong Ba Nhat, Ky Pham Van, Hung Le Xuan, Umberto D'Alessandro, Annette Erhart

**Affiliations:** 1 Institute of Tropical Medicine, Nationalestraat 155, 2000 Antwerp, Belgium; 2 Partners for Applied Social Sciences | PASS International, 3980 Tessenderlo, Belgium; 3 National Institute for Malariology, Parasitology and Entomology, Luong The Vinh Street 245, Hanoi, Vietnam; 4 Provincial Malaria Station. Ngo Gia Tu street 156 - Phan Rang City. Ninh Thuan Province, Vietnam

## Abstract

**Background:**

Despite Vietnam's success in reducing malaria mortality and morbidity over the last decade, malaria persists in the forested and mountainous areas of the central and southern provinces, where more than 50% of the clinical cases and 90% of severe cases and malaria deaths occur.

**Methods:**

Between July 2005 and September 2006, a multi-method study, triangulating a malariometric cross-sectional survey and qualitative data from focused ethnography, was carried out among the Ra-glai ethnic minority in the hilly forested areas of south-central Vietnam.

**Results:**

Despite the relatively high malaria burden among the Ra-glai and their general awareness that mosquitoes can transmit an unspecific kind of fever (84.2%), the use of bed nets, distributed free of charge by the national malaria control programme, remains low at the farmers' forest fields where the malaria risk is the highest. However, to meet work requirements during the labour intensive malaria transmission and rainy season, Ra-glai farmers combine living in government supported villages along the road with a second home or shelter at their slash and burn fields located in the forest. Bed net use was 84.6% in the villages but only 52.9% at the forest fields; 20.6% of the respondents slept unprotected in both places. Such low use may be explained by the low perception of the risk for malaria, decreasing the perceived need to sleep protected. Several reasons may account for this: (1) only 15.6% acknowledged the higher risk of contracting malaria in the forest than in the village; (2) perceived mosquito biting times only partially coincided with *Anopheles dirus ss *and *Anopheles minimus A *true biting times; (3) the disease locally *identified *as 'malaria' was hardly perceived as having an impact on forest farmers' daily lives as they were unaware of the specific kind of fevers from which they had suffered even after being diagnosed with malaria at the health centre (20.9%).

**Conclusions:**

The progressive confinement of malaria to minority groups and settings in the Greater Mekong sub-region implies that further success in malaria control will be linked to research into these specific socio-cultural contexts. Findings highlight the need for context sensitive malaria control policies; not only to reduce the local malaria burden but also to minimize the risk of malaria spreading to other areas where transmission has virtually ceased.

## Background

Despite Vietnam's success in reducing malaria mortality and morbidity over the last decade, malaria persists in the forested and mountainous regions of Vietnam's central and southern provinces where more than 50% of the clinical cases and more than 90% of severe cases and malaria deaths occur [1-4]. This progressive confinement of the disease to specific areas and risk populations over the past years has altered the characteristics of malaria epidemiology in Vietnam, now affecting especially poor ethnic minorities in remote areas, forest workers and migrants [3,4]. Such a shift from majority society to minority groups and settings is also taking place in other regions of the Greater Mekong Sub-region (GMS), as shown by the available data from Cambodia and Laos [5]. Therefore, further success in malaria control will be linked to a better understanding of these minority groups and settings, and not only to reduce the local malaria burden but also to minimize the risk of spreading malaria to other areas where transmission has virtually ceased [6].

One of the keystones of Vietnam's malaria control programme among ethnic minorities and in remote rural areas has been the free-of-charge distribution and insecticide-treatment of bed nets. However, despite their proven efficacy and effectiveness, there is ample evidence indicating that they are not always locally accepted, practical to use or easily accessible. A major factor generally limiting bed nets' acceptability and accessibility is their high price in relation to the financial capacities of people in the communities [7-10]. However, since, in the present context bed nets are distributed free-of-charge, other constraints must be considered.

The present research was conducted among the Ra-glai ethnic minority in south-central Vietnam with the aim of gaining insight into exposure to malaria infection in relation to bed net use and the perceived risk of infection.

## Methods

### Study site and population

The study was carried out in 10 communities (~20,000 people) situated in the hilly forested areas of the Bac Ai and Ninh Son districts of Ninh Thuan Province in south-central Vietnam in the framework of a community-based, cluster randomized trial assessing the effectiveness of long-lasting insecticidal hammocks (LLIH) in controlling forest malaria [6]. Malaria transmission is perennial with two peaks (May-June and October-November), at the start and at the end of the rainy season. In a survey carried out in November 2003, the overall parasite rate was 13% (up to 40% in some villages) and the prevalence of anti-malarial antibodies 37% (up to 75% in some villages) [11]. Malaria control is based on early diagnosis and treatment with artemisinin-based combination therapy and the provision of insecticide-treated nets (ITNs), distributed free-of-charge and insecticide-treated twice a year by the national malaria control programme. Reported ITN use in the study area was high (86.3%, in April 2004) with a median coverage of 2.5 people per bed net [12]. Similar to other forested areas of central Vietnam, despite intensive efforts, malaria remains difficult to control due to the complex interactions between humans, vectors and environmental factors [4].

The Ra-glai, or '*people of the forest*' in Ra-glai and Cham languages [13,14], are of Malayo-Polynesian descent and represented 86% of the study population [11]. Linguistic evidence suggests that they are possibly descendents of coastal *Cham *that were historically pushed inland to the forested and mountainous regions [15,16]. Today, the Ra-glai are a largely impoverished ethnic minority in Vietnam, almost exclusively dedicated to small-scale subsistence slash and burn agriculture in fields located in the surrounding forests. According to a survey carried out in 2003, 80% of the active population can be categorized as 'forest worker', 99.5% of which participate in slash and burn agriculture, occasionally combined with hunting, gathering of forest products and logging [12]. The Ra-glai's heavy dependence on the forest for subsistence activities places them at greater risk for malaria infection, a risk that is further increased by staying overnight in the forest [12,17]. Indeed the main malaria vector *Anopheles dirus sensu stricto*, is a sylvatic and highly anthropophilic species, resting mainly outdoors and biting early in the evening [18].

### Research strategy

The implemented research strategy was based on methodological triangulation (the combination of various methods and data sources to refine and test particular interpretations and hypotheses), combining both qualitative and quantitative methods in order to limit bias and enhance the strengths of the respective research methods [19,20]. The combination of both qualitative and quantitative methods during fieldwork allows for the confirmation of specific patterns while also facilitating the detection of new and unexpected variables. Methodological triangulation has the additional advantage of being able to contextualize trial data through qualitative sampling techniques and data analysis, and of facilitating the quantification of qualitative variables from focused ethnography through random sampling and statistical analysis.

### Data collection

#### Qualitative data

Field work was carried out during three field stays of approximately one month each, between July 2005 and September 2006. The aim of the stays was to provide contextualized data on the study area and population, to refine existing research questions and to detect new potential risk factors for malaria infection. During fieldwork, 12 villages were selected for their theoretical relevance, representing seven communes distributed in the two study districts. A total of 101 Ra-glai households were included: 58 households (57.4%) were informants selected during participant observation at public spaces (forest fields, roads to and from the forest, local shops and community health centres), while an additional 43 households (42.6%) were selected due to the recent malaria episode of one of their household members. The latter were identified by passive case detection (PCD) between August 1 and October 15, 2005, and represent all malaria cases identified during that period in their respective villages, and 68.3% (total = 63) of those identified in the five communities belonging to Bac Ai District. Additional interviews were held with health staff, including Ra-glai Hamlet Health Workers (HHWs) and general health staff from the seven CHCs and different institutions, including the Ninh Thuan Provincial Malaria Station (PMS), the National Institute of Malariology, Parasitology & Entomology (NIMPE) Hanoi and the Institute for Tropical Medicine Antwerp (ITM).

##### Participant observation

Participant observation consisted of participating in everyday activities, observing events in their usual context and carrying out reiterated informal conversations and interviews in order to build up confidence with informants and understand more sensitive subjects, such as forest activities, some of which are illegal. This technique proved useful in identifying bias in overall response during interviews and surveys.

##### In-depth interviews

One to five open or semi-structured formal and/or informal interviews were held with all included households (total = 101) during fieldwork. Households and key informants were contacted and interviewed reiteratively during the same or different field stays, building up confidence between researchers and respondents, which played a key role in distinguishing between biased and genuine response.

##### Focus group discussions

Several attempts were made to organize focus group discussions with local health staff but this technique was abandoned as the discussions tended to reflect the groups' internal hierarchical organization and adherence to social order rather than the aimed diversity of opinions and knowledge.

#### Quantitative data

During the follow-up survey carried out in December 2005 (hereafter "2005-survey"), a questionnaire was administered to 3,685 randomly selected Ra-glai individuals surveyed bi-annually within the LLIH study [6]. A total of 20 questions were asked as part of a standardised pre-coded questionnaire specifically related to the Ra-glai house-settlement system, forest activities, sleeping habits and knowledge and practices related to malaria exposure. The estimation of bed net use in homes and plot huts at forest fields was done on the sub-sample of 635 respondents that stated having slept at their fields in the month prior to the survey (17.2% of the total survey respondents).

### Mosquito collection

Mosquito collections were carried out in three villages in Ma Noi commune and five villages belonging to Phuoc Binh commune. In total, five surveys were included (Nov 2004, Sept/Oct 2005, Nov/Dec 2005 & 2006). Human outdoor landing collections were done inside the villages (subsequently called 'village') and near forest shelters ('forest') for eight nights per survey in each location. Mosquitoes were collected from 18.00 until 06.00, stored by hour and morphologically identified in the field using a standardised key for medically important anophelines in Southeast Asia (for more details please refer to [21]).

### Data analysis

In accordance with the research strategy, data analysis was a continuous, flexible and iterative process: preliminary data from different techniques were collected and analysed; further research was then conducted confirming or refuting temporary results until saturation was reached and the data theoretically supported. Qualitative data were systemised and analysed with N/Vivo Qualitative Analysis software (QSR International Pty Ltd. Cardigan UK). Percentages presented as a result from qualitative data analysis are illustrative of field research, hence not based on random sampling and should, therefore, not be equated to survey data.

Survey data were double entered and cleaned in Epi Info 6.04 (CDC, Atlanta; WHO, Geneva 1996), and analysed in STATA 9.0 software (Stata Corp., College Station, TX). Descriptive statistics were computed using the "svy" command in STATA, in order to take into account the survey characteristics. During the last phase of the fieldwork, preliminary results of the 2005-survey were analysed and further qualitative research was conducted to acquire supplementary data for the interpretation of those results.

### Concept definitions

In accordance with existing social science theory [22], risk perception for suffering from malaria was operationalized as encompassing the following components: (i) the perceived exposure to the disease, (ii) the perceived susceptibility to malaria infection and (iii) the perceived severity of malaria.

### Ethical considerations

The study was approved by the ethical committees of the Institute of Tropical Medicine, Antwerp, Belgium and the NIMPE, Hanoi, Vietnam. During field work, all interviewers followed the Code of Ethics of the American Anthropological Association (AAA) [23]. As proposed by the AAA, all interviewees were informed before the start of the interview about project goals, the topic and type of questions, their right to refuse being interviewed, to interrupt the conversation at any time, and to withdraw any given information during or after the interview, and the intended use of the results for scientific publications and reports to health authorities. Oral consent was preferred since the interviewees were not put at any risk of being harmed physically or psychologically and because the act of signing one's name when providing information during informal conversations could be a potential reason for mistrust [24].

## Results

### Residence patterns

To meet work requirements during the labour intensive malaria transmission and rainy season, Ra-glai slash and burn farmers combined living in government-supported villages along the road with a second home or shelter at their slash and burn fields located in the forest. The large majority of Ra-glai families spent nights at their fields, particularly during harvest times, among other reasons, so as not to lose time travelling to and from their fields and in order to protect their harvests from rodents, cattle and other animals.

### Bed net use

During fieldwork, bed net use was observed to be irregular both in villages and at forest fields. Even when bed nets were used, not all members of the household necessarily slept under one, and the following main constraints were reported: (i) the bed net can be cast aside when it is too hot at night; (ii) the use of bed nets increases the distance to the fire when it is cold and humid; (iii) or, without this precaution, leads to the fear that the net will catch fire while sleeping; (iv) poorer families often use the nets to cover themselves due to a lack of means to buy blankets; and, (v) nets are often damaged when living conditions are poor and people can not afford to purchase extra ones.

Furthermore, the combination of sleeping at fields and in villages leads to complex mobility patterns affecting bed net use. While the entire family moves together for longer periods, one or more household members might be required to sleep sporadically in the other location (field/village). Some examples of household members sleeping in the village while the rest of the family remains at the field are: children attending school and adults bringing products to villages to sell, buying goods in local shops or visiting relatives. To ensure coverage, ITNs would, therefore, have to be regularly transported between villages and fields, a cumbersome undertaking (which in practice is not done), and that poses the additional problem of leaving the household members who stay behind exposed.

According to the 2005-survey, 84.6% of respondents were regularly using bed nets in their villages (Table 1). However, among Ra-glai farmers sleeping in the forest, only about half (52.9%) reported sleeping under a bed net at the forest fields; only 41.6% reported regularly using a bed net both in the village *and *at their forest fields, while 20.6% slept unprotected in both places.

**Table 1:** Bed net use among the Ra-glai (2005-survey)

	n (%)
**Bed net use in the village among all survey participants (n = 3,685)**	
- Do not use	332 (9.0%)
- Sometimes	237 (6.4%)
- Usually ("protected")	3,116 (84.6%)
	
**Bed net use in the village and at fields among people sleeping in the forest (n = 635)**	
- People sleeping protected in the village	430 (67.7)
- People sleeping protected at the fields	336 (52.9)
- People sleeping unprotected in village and in fields	131 (20.6)
- People sleeping protected both in village and at fields	264 (41.6)

### Aetiology and knowledge of malaria

#### Knowledge of malaria

About half (50.5%) of interview respondents were unsure what exactly the term "malaria" -"*sot ret*" (literally 'hot fever') in Vietnamese or "*Lah Sakih*" in Ra-glai-referred to (Table 2). Furthermore, knowledge about the illness identified as malaria, when present, was often inconsistent with biomedical notions:

**Table 2:** Field work data on malaria awareness among all Ra-glai informants (n = 101) and among those having been identified with malaria one month prior to the interview (n = 43)

	n (%)
**Respondents acknowledging that mosquitoes transmit fever (n = 101)**	
- Yes	85 (84.2%)
- No	16 (15.8%)
	
**Respondents acknowledging 'malaria' ('sot ret'; 'lah sakih') (n = 101)**	
- Yes	50 (49.5%)
- No	51 (50.5%)
	
**Did you ever have fever due to mosquito bites? (n = 43 malaria patients)**	
- No	7 (16.3%)
- Yes	19 (44.2%)
- Don't know	17 (39.5%)
	
**What kind of fever/disease were you treated for at the CHC? (n = 43)**	
- Malaria	9 (20.9%)
- "I don't know" & Other	34 (79.1%)

"*I can't get the fever because I have a lack of red blood cells*" (Ra-glai woman. New Bo Lang);

"*People get malaria because they eat food that's not safe, sleep without a bed net or have their houses dirty*" (Ra-glai secondary school student. Ma Ty, Phuoc Tan).

#### Cause of fever

Of the 145 respondents stating to have suffered from malaria in the month prior to the 2005-survey, only 29.6% attributed it to mosquito bites (Table 3). More than half (55.2%) did not mention any cause and 15.2% cited other reasons, such as excess of sun exposure or hard work.

**Table 3:** Malaria awareness among Ra glai participants in the 2005-survey

Total Ra-glai participants = 3,685	n (%)
**Did you have fever during the past month? (n = 3,685)**	
- Yes	1,021(27.7%)
- No	2,656 (72.1%)
- Don't know	8 (0.2%)
	
**Kind of fever? (n = 1,021)**	
- Malaria	144 (14.1%)
- Flu	354 (34.7%)
- Other (headache, tonsillitis, bronchitis, gastritis, etc)	54 (5.3%)
- Don't know	448 (43.9%)
- Missing	23 (2.1)
	
**What was the cause of fever? (n = 1,021)**	
- Mosquito bites	81 (7.9%)
- Bad food	14 (1.4%)
- Bad weather	96 (9.4%)
- Too much work	13 (1.3%)
- Don't know	761 (74.5%)
- Missing	55 (5.4%)
	
**What was the cause of your malaria episode? (n = 145)**	
- Mosquito bite	43 (29.60%)
- "I don't know"	80 (55.2%)
- Other causes	22 (15.2%)
	
**Did you ever have fever due to mosquito bites? (n = 3,685)**	
- Yes	1,327 (36.0%)
- No	1,834 (49.8%)
- Don't know	504 (13.7%)
- Missing	20 (0.5%)
	
**At what time do mosquitoes bite in the village? **(median) **(n = 3,685)**	18 to 20 h
	
**At what time do mosquitoes bite in the forest fields? **(median) **(n = 3,685)**	06 to 10 h
	
**Mosquitoes are more dangerous for malaria infection? (n = 3,685)**	
- In the village	549(14.9%)
-In forest fields	575 (15.6%)
- Same	218 (5.9%)
-Don't know	2,288 (62.1%)
- Missing	55 (1.5%)

#### The fever-mosquito link and malaria infection

The general link between unspecified types of fever and mosquito bites, when elicited during field work, was acknowledged by 84.2% of Ra-glai households (Table 2), presumably thanks to IEC campaigns on malaria control in the area. However, in practice, the general knowledge of this link did not readily translate into people's awareness of their own vulnerability to malaria. This was confirmed in the quantitative data by the low proportion of survey respondents (29.6%) that attributed their last malaria episode to mosquito bites (Table 3).

### Perceived severity

The perceived severity of malaria was not directly followed up in the 2005-survey because local Ra-glai concepts could not be directly equated to 'malaria'. However, it is interesting to note that among previously confirmed malaria patients followed-up during field work (n = 43), only one fifth actually knew they had been treated for malaria (Table 2).

### Perceived susceptibility to malaria infection

#### Perception of exposure at fields

15.6% of survey participants considered mosquitoes in the forest fields to be more dangerous for malaria infection than in the village (Table 3). When available, local knowledge on vector control and malaria transmission incorporated various elements from official public health campaigns. Nevertheless, the message often remained disjointed as illustrated in the following statements:

"*Mosquitoes usually bite during the daytime. (...) I light a fire to drive them away, but the mosquitoes in the village are not afraid of the smoke!*" (Ra-glai man. Da Trang);

"*The mosquitoes in the village are more dangerous because there is electricity in the village and so they appear. And in my plot hut there is no electricity, so there are only a few mosquitoes*" (Ra-glai forest farmer. Ma Ty, Phuoc Thang).

*"There are many mosquitoes here but the mosquitoes at the forest field don't cause malaria. The ones in the village do because there is stagnant water around the house*" (Ra-glai farmer, Ta Lot);

"*In the village mosquitoes are more dangerous than in the forest because there are many sick people in the village. The mosquitoes bite these people and then transmit their diseases to the others*" (Ra-glai forest farmer. Ma Ty, Phuoc Thang).

Furthermore, outdoor human landing collections showed that, though the mean number of bites per man-night (BMN) for all Anopheles mosquitoes was higher in the forest fields (4.3 BMN) than in the village (2.5 BMN), there was no difference when considering all mosquito species together (*Culicidae*), (20.7 and 22. BMN, respectively) (Wim van Bortel, unpublished data); therefore, the perceived mosquito nuisance was not greater in the forest field than in the village.

#### Mosquito biting times

**Figure 1:**
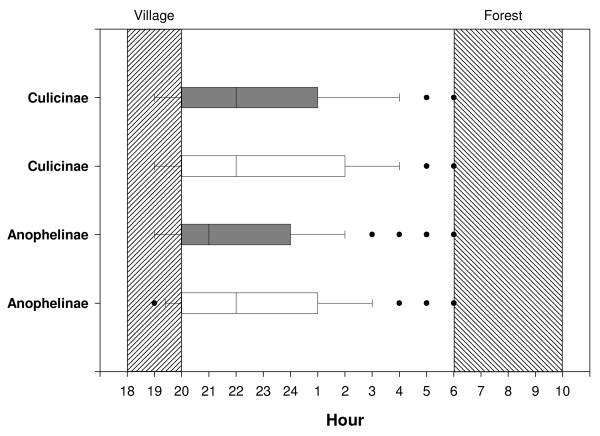
**Outdoor human landing collections in the forest and in the village by collection hour**. White box plot: mosquito biting activity in Forest. Black box plot: mosquito biting activity in the village (Wim Van Bortel, unpublished data). The perceived median biting time in the village and the forest is indicated by the hatched areas.

Inquiring about the perceived mosquito biting times, survey participants were asked at what time mosquitoes bite in the village and at the forest fields (Table 3). The median biting time for mosquitoes in the village was between 18:00 and 20:00, while at the forest fields this was from 06:00 until 10:00 in the morning. Qualitative data further confirmed that respondents were aware of the risk of mosquito bites in the evening and early morning, which corresponded to the times when insects were more visible and a greater nuisance, but that they were commonly under the impression that few to no mosquitoes were present for the rest of the night. According to entomological data (Figure 1), biting was indeed early, with 50% of all bites occurring before 22.00 h. Nevertheless, the perceived median biting time in villages (18.00-20.00) represents only around 25% of all bites per night, and the stated time frame for mosquito biting at forest fields (06.00-10.00) corresponds more to other insect activity than to mosquitoes who become largely inactive after sunrise.

## Discussion

In this multi-method study, a particular emphasis was placed on the perceived drawbacks/benefits of bed net use in relation to the perceived risk for suffering from malaria and the awareness of exposure at forest fields.

### Residence and mobility patterns

The unexpected residence patterns (village/fields) among the Ra-glai lead to a re-evaluation of bed net use, now including both village homes and field shelters, and clearly highlight that minority settings require additional research and adapted research methods. In the present context, the understanding of local residence patterns was fundamental for the assessment of perceived risk to malaria, and could only be properly evaluated through methodological triangulation, including participant observation.

### Perceived level of exposure

The extent to which people believe themselves to be at risk for malaria infection depends on their perceived level of exposure to the disease. Despite a general awareness that mosquitoes transmit unspecific types of fever (84.2%), only about half of the survey respondents reported regular bed net use at their fields, where the malaria risk is the highest. And only 15.7% of the population was aware of the greater risk of contracting malaria in the forest. This can be understood in relation to the generally perceived mosquito nuisance. Despite a higher number of Anopheles bites per man-night in the forest, as shown by entomological results from the study area, there was no difference between forest and village in terms of mosquito bites when accounting for all species. Therefore, the susceptibility to mosquito biting does not vary between forest and village, rendering a higher perceived risk for malaria at the forest field understandably negligible.

Another component influencing exposure and reducing the perceived need to use bed nets at night is the reasonable perception that mosquito biting times coincide with those when insects are a greater nuisance, i.e. early morning and at sunset, although this period only represents a small portion of the actual biting times.

### Perceived susceptibility and severity

Studies have shown that malaria is often not perceived as severe, but rather as a mild, self-limiting illness [22]. In the study area, the low susceptibility to malaria is manifest in the fact that (i) only half of the population was actually able to identify the illness by its name (in Vietnamese or in Ra-glai language); (ii) 75% of survey respondents were unaware of the cause of their last fever; and, (iii) diagnosed malaria patients were largely unaware of the specific kind of fever they had suffered from. Such lack of awareness about having malaria, even after health centre diagnosis, illustrates the minimal perceived severity of this disease. At the same time, the general knowledge that mosquitoes transmit an unspecified kind of fever, in practice, did not translate into people's awareness of their own vulnerability. Malaria, therefore, had little *perceived *influence on people's daily lives. This lack of association between malaria and risk reduces the perceived need to prevent mosquito bites and to use bed nets.

### Benefits and drawbacks of bed net use

It is against this background that the potential benefits and drawbacks of bed net use must be understood. Worldwide, commonly reported barriers to bed net use are found in people's feeling of being suffocated under the bed nets, the heat, and a sense of confinement while under the bed nets [25]. The colour of bed nets can also deter people from using them, e.g. in Burkina Faso, the white colour of nets is associated with funeral rites. The mesh size of the bed net can also affect its use. In the Peruvian Amazon, the standard ITNs' and LLINs' mesh size is often considered too transparent to allow for privacy [26,27]. Notably, though the perceived benefits of sleeping under a bed net can be related to prevention of malaria or other vector borne diseases, they often seem to correspond more to supplementary benefits of bed net use, such as avoiding the nuisance of mosquitoes and other insects while sleeping. These perceived benefits have been shown in numerous studies and contexts [28-32]. Examples include general protection against insects, the provision of warmth and intimacy, as reported in Nigeria [31] and Ghana [32], and providing an inside/outside division when houses have no outside walls, as is the case in the Peruvian Amazonian region [26,33].

Where mosquito nets are commonly used, the complementary benefits often appear to be more decisive in fostering their use than malaria prevention itself. Therefore, convincing people to use bed nets will be more difficult in regions, contexts and periods where no extra perceived benefits are apparent. Among the Ra-glai in the study area, there seems to be few perceived direct or complementary benefits of bed net use at forest fields. There is a low level of perceived exposure, susceptibility and risk associated with malaria infection and, therefore, while the Ra-glai value the use of bed nets, there is little incentive for them to do so continuously. Moreover, various constraints were reported, such as the difficulty of using them while sleeping close to a fire at night for warmth and practical factors associated with mobility patterns.

## Conclusion

This study clearly highlights that improving bed net use in a minority setting requires research into the local social and cultural context as well as the use of adapted research methods. Furthermore, while it seems straightforward to conclude that improving IEC and strengthening other health system factors, such as the communication during the health encounter upon malaria diagnosis, are required to create more awareness on the risk of malaria in this context, additional poverty-related constraints and the requirements of local mobility patterns have to be considered as they might undermine the effectiveness of public health messages. The challenge, therefore, consists of finding context sensitive malaria control strategies that incorporate both health system and structural factors, as conventional health education messages and prevention strategies, even when locally accepted, can not always be translated into daily practice.

## Competing interests

The authors declare that they have no competing interests.

## Authors' contributions

The focused ethnography was designed by KPG and JMR and carried out by KPG, XNX and TBN. AE contributed to the study design, study coordination and supervision, field work, statistical analysis and revision of the manuscript. TND contributed to the study design, study coordination and supervision, survey data entry, cleaning and analysis. KPV was responsible for the coordination and supervision of all field activities and field staff. HLX contributed to the study design, study coordination and supervision, and reviewed the manuscript. TLK contributed to the study design and reviewed the manuscript. WVB offered unpublished BMN data proceeding from outdoor mosquito collection in the study area. UD contributed to the study design, study coordination and supervision, survey data analysis and revision of the manuscript. All authors have read and approved the final manuscript.
